# Risk factors for intimate partner emotional violence among women in union in Uganda

**DOI:** 10.3389/fsoc.2023.840154

**Published:** 2023-05-05

**Authors:** Resty Nakitto, Abel Nzabona, Stephen Ojiambo Wandera

**Affiliations:** ^1^Department of Population Studies, School of Statistics and Planning, College of Business and Management Sciences, Makerere University, Kampala, Uganda; ^2^Centre for Basic Research, Kampala, Uganda

**Keywords:** intimate, emotional, alcohol, controlling behavior, consumption, violence

## Abstract

**Introduction:**

Despite the growing evidence of the prevalence of gender-based violence in Uganda, less is known about the factors influencing intimate partner emotional violence (IPEV) among married women in the country. This study investigated the social demographic factors associated with IPEV among married women aged 15 years and older.

**Data and methods:**

The study used the 2016 Uganda Demographic Healthy Survey (UDHS) data. A weighted sample of 5,642 women who had been in a union was selected. A binary logistic regression model was fitted to analyze the predictors of IPEV.

**Results:**

Almost four in 10 (38%) married women experienced IPEV. Witnessing parental violence (OR = 1.37, CI = 0.59–0.92), partner's controlling behavior (OR = 4.26, CI = 3.29–5.52), and attaining age 35+ (OR = 1.44, CI = 1.06–1.95) increased the odds of IPEV. Residing in rural areas (OR = 0.004, CI = 0.48–0.99) and having higher education (OR = 0.51, CI = 0.26–1.00) decreased the odds of IPEV.

**Conclusion and implications:**

Witnessing parental violence, alcohol consumption, age, place of residence, partner's controlling behavior, and level of education influence IPEV among married women in Uganda. The findings have several implications including strengthening IPEV-prevention campaigns, women empowerment, and alcohol consumption regulations.

## Introduction

Intimate Partner Emotional Violence (IPEV) is any behavior within an intimate relationship that causes physical, sexual, or psychological harm (Onanubi et al., 2017). The acts of emotional violence include verbal assault, dominance, control, isolation, ridicule, or the use of intimate knowledge for dilapidation (Follingstad et al., 2005). Engel (2002) adds that emotional abuse can also mean any non-physical behavior or attitude that control, subdue, punish, or isolate another person through the use of humiliation or fear. Perpetrators of emotional violence engage in acts that include humiliating the victim, controlling what the victim can or cannot do, withholding information from the victim, deliberately doing something to make the victim feel diminished or embarrassed, isolating the victim from friends and/or family, denying the victim access to money or other basic resources, stalking the victim, demeaning the victim in public or in private, and undermining the victim's confidence and/or sense of self-worth (O'Leary, 2004). A person experiencing IPEV can witness various consequences which are not limited to suicidal ideation, depression, and posttraumatic stress disorders such as intense fear, shortness of breath, nightmares, sleeping difficulties, dizziness, cramps, and many more (Soomar, 2015). All these effects if untreated can expose the victim to mental illness and death in the long run.

Globally, 48.4% of women and 48.8% of men have experienced at least one psychologically aggressive behavior by an intimate partner. Four in 10 women and men have experienced at least one form of coercive control by an intimate partner in their lifetime. Approximately 18.7% of women have experienced threats of physical harm by an intimate partner and women who earn 65% or more of their household's income are more likely to be psychologically abused by their intimate partners (National Coalition Against Domestic Violence, 2015). These percentages remain alarmingly high with short- and long-term effects on women's health (Lawoko et al., 2013). Reports show that more than half of the victims of IPEV often experience short-term and long-term effects which include depression, post-traumatic stress disorder, suicidal ideation, low self-esteem, and difficulty trusting others (National Coalition Against Domestic Violence, 2015).

In Uganda, the 2016 Uganda Demographic and Health Survey (UDHS) reveal that 56% of the women had experienced at least one form of violence including emotional, physical, or sexual. That was far away from the fifth Sustainable Development Goal (SDG) which targets ending all forms of discrimination and violence against women and girls (Loewe and Rippin, 2015). Despite the growing understanding of IPEV as an important public health and safety issue, its complete eradication was still challenging for several reasons such as a lack of good data on the nature and magnitude of IPEV, limited funding and resources to address it, preservation of cultural norms and practices, and the long-held assumptions that violence is inevitable and preventable.

Intimate partner emotional violence is a risk factor for various adverse psychological health outcomes and is a major public health issue with short-term and long-term effects, for example, the risk of contracting HIV and STIs, pregnancy complications, miscarriages, low birth weight, and so many others (Uwayo, 2014). Only one-third (29%) of women exposed to violence are able to receive primary healthcare (Devries et al., 2013). These risks have directed the international community to implement laws and measures that would protect women from gender-based violence including IPEV. However, the existence of these international initiatives which include laws and policies in the country had not been able to eliminate IPEV completely.

Existing studies had focused on physical violence, sexual intimate partner violence, non-partner sexual violence, and the effect of violence on child growth (Whitaker, 2014; Durevall and Lindskog, 2015; Mönttinen and Tetri, 2016; Cools and Kotsadam, 2017). Other studies had focused on the attitude of men and women toward IPV and the relationship between IPV and the contraction of HIV and other sexually transmitted diseases (Capaldi et al., 2012; Wagman et al., 2015). However, limited research had been conducted about factors influencing intimate partner emotional violence among married women in Uganda.

## Conceptual framework

**Figure 1:**
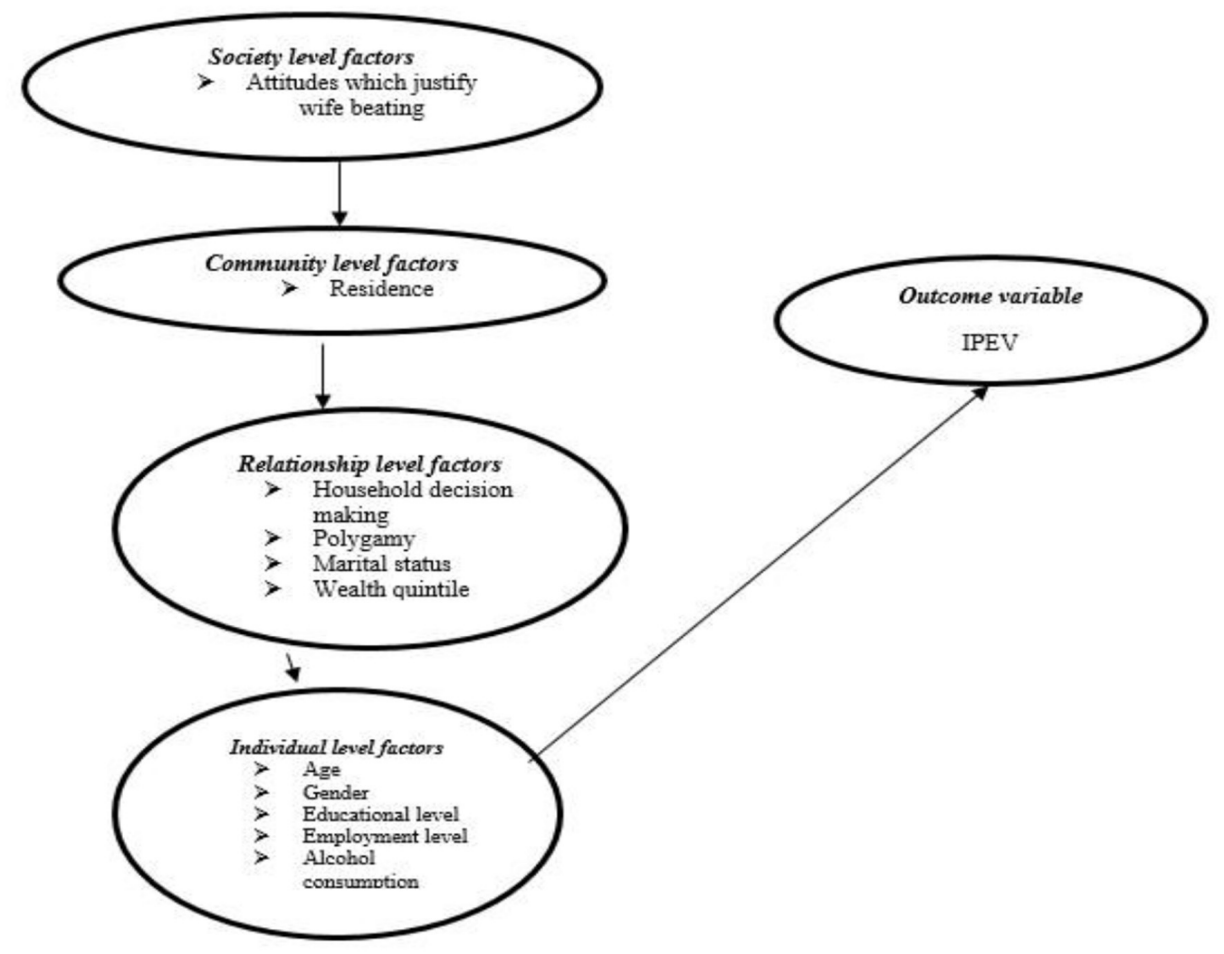
Conceptual framework for the study of IPEV. Adapted from Heise (1998).

The conceptual framework was developed from an integrated ecological model to explain the factors which influence violence. Figure 1 shows an IPEV conceptual framework adapted from Heise (1998). Four levels of the sources of violence were defined, namely, society, individual, relationship, and community level factors (Azam and Naylor, 2013). Individual-associated factors are factors within the individual that may be biological and could increase the likelihood of being a victim or perpetrator of IPEV. They include gender, age, level of education, alcohol consumption, witnessing parental violence, and employment levels (Azam and Naylor, 2013).

Relationship level factors are those which involved close social interactions between the individual and the people in his or her immediate environment. Those most common include the number of wives, ability to make household decisions, control of resources, and wealth quintile. The model further presupposed that community-level factors referred to the community contexts within which violence occurs. In the study, the residence is the community-level factor that was put into consideration (Azam and Naylor, 2013).

Finally, the society-level factors are related to the systems of the society and culture where the person lived. This meant that men were exposed to cultural messages which encouraged male superiority and granted them the right to control female behavior. The society-level factors considered in the study are attitudes that justify wife beating. Conclusively, no single factor works in isolation. All factors should be tackled simultaneously if the issue of violence is to be addressed.

## Data and methods

### Data source

The study used secondary data from the 2016 Uganda Demographic and Health Survey (UDHS). It covered all regions and districts in the country. The sampling frame used for the 2016 UDHS was the frame of the Uganda National Population and Housing Census (NPHC), conducted in 2014; the sampling frame was provided by the Uganda Bureau of Statistics. The census frame is a complete list of all census Enumeration Areas (EAs) created for the 2014 NPHC. In Uganda, an EA is a geographic area that covers an average of 130 households (Uganda Bureau of Statistics (UBOS) ICF, 2018).

## Survey design

The 2016 UDHS sample was stratified and selected in two stages. In the first stage, 697 EAs were selected from the 2014 Uganda NPHC: 162 EAs in urban areas and 535 in rural areas. Households constituted the second stage of sampling. A listing of households was compiled in each of the 696 accessible selected EAs from April to October 2016. The sample EAs were selected independently from each stratum using probability proportional to size. The 20,880 selected households resulted in 18,506 women successfully being interviewed, with an average of 1,200 complete interviews per domain [Uganda Bureau of Statistics (UBOS) ICF, 2018]. In addition, all women aged 15–49 years who were either permanent residents of the selected households or visitors who stayed in the household the night before the survey were eligible to be interviewed. In the survey, interviewers used tablet computers to record all questionnaire responses during the interviews. The tablet computers were equipped with Bluetooth technology to enable remote electronic transfer of files, such as assignments from the team supervisor to the interviewers, individual questionnaires among survey team members, and completed questionnaires from interviewers to team supervisors. The CAPI data collection system employed in the 2016 UDHS was developed by the DHS Program with the mobile version of CSPro. The CSPro software was developed jointly by the U.S. Census Bureau, Serpro S.A., and The DHS Program [Uganda Bureau of Statistics (UBOS) ICF, 2018].

### Validation of survey instruments

The UDHS technical team, composed of staff from UBOS and ICF, participated in a 2-day training of trainers (TOT) workshop conducted on 17 and 18 March 2016. The pretest training took place between 21 March and 8 April 2016 at the Imperial Golf View Hotel in Entebbe Municipality. The UDHS technical team and ICF technical specialists trained 45 participants to administer the paper and electronic questionnaires. The pretest fieldwork took place between 13 and 15 April 2016 in clusters surrounding the training venue in Entebbe Municipality that were not included in the 2016 UDHS sample area, which covered ~240 households. The UDHS technical team and ICF conducted debriefing sessions with the pretest field staff on 16 April 2016; modifications to the questionnaires were made based on lessons learned from the exercise. Teams then spent an additional week upcountry testing the translations.

### Study population

The study only focused on married women aged 15–49 years. In the survey, 9,232 women were interviewed. From this survey, a weighted sample of 5,642 women who reported being union or cohabiting for the last 12 months was extracted. The domestic violence weighting (d005) was applied to attach the weights during analysis.

### Variables

The outcome variable of the study was Intimate Partner Emotional Violence (IPEV). In the UDHS, information about IPEV was captured by asking a question like Did your husband/partner ever:

- Say or do something to humiliate you?
- Threaten to hurt or harm you or someone you care about?
- Insult or make you feel bad about yourself?

The outcome variable was binary because, for each of the above questions, the respondent was expected to answer either Yes or No. The binary response from the three questions was merged into an aggregate measure of intimate partner emotional violence (variable d104) in the UDHS. The outcome variable was coded 0 = No (did not experience IPEV) and 1 = Yes (experienced IPEV).

The explanatory variables of the study were classified into three categories, namely, women's social demographic factors which included the following: number of other wives, marital status, witnessed parental violence, age, residence, sex of household head, wealth index, education level of the wife and husband, and type of earnings. Others were women empowerment indicators which included participation in decision-making and ownership of assets. The last set comprised of partners' controlling behavior and attitudes justifying physical violence and alcohol consumption.

Social demographic factors were measured by whether the husband had one or more co-wives = 1 and no other wives = 2, current marital status (which is married or cohabiting), and witnessed parental violence which was measured by whether the respondent reported ever witnessing her father beating her mother (with a binary outcome of 0 = No, 1 = Yes); age was categorized into three groups (15–24, 25–34, and 35+years), residence (urban = 1 and rural = 2), sex of the household head (male = 1 and female = 2), wealth index, education level of the wife and husband, and type of earnings.

Second, are women's empowerment indicators which include participation in decision-making autonomy regarding who usually makes decisions about (a) how women's earnings are used; (b) women's healthcare; (c) large household purchases; (d) visits to family or relatives; and (e) what to do with the money the partner earns. Responses to these questions were recorded into two categories (1 = woman decides alone/jointly with partner, 0 = partner alone/others). The responses were further merged where participation in any of the above decisions was coded 1 = Yes and lack of participation 0 = No. The assumption was that women who made decisions either alone or jointly with their partners were more empowered than those in households where decisions were made by either their partners alone or other people. Ownership of a house or land was recorded into two categories: woman alone/jointly with the partner as the empowered category and partner alone/others as the other.

The partner controlling behavior of men where women were asked whether their present partners: (a) were jealous if respondents talked with other men; (b) accused them of unfaithfulness; (c) did not permit them to meet female friends; (d) tried to limit respondents' contact with family, and (e) insisted on knowing where they were. Attitudes justifying physical violence were measured by questions concerning whether wife beating was justifiable if the wife: (a) goes out without telling her partner; (b) neglects their children; (c) argues with her partner, and (d) refuses to have sex with her partner. A positive response to any of the above was (1 = “yes” or 0 = “no”). All these variables had binary responses (0 = no and 1 = yes).

Partner's alcohol consumption was measured by two questions: (a) Does your partner drink alcohol? This was coded as a binary outcome (0 = No, 1 = Yes). The second follow-up question was asked to those who said yes to drinking alcohol: (b) How often does (did) he get drunk: often, only sometimes, or never? The response categories were 0 = never, 1 = often, 2 = sometimes. I also included women's attitudes toward their partners—whether they were afraid of their partners—in this category of variables. Women were asked if they were afraid of their partners. This was categorized as 0 = never, 1 = most of the time.

### Data analysis

Data analysis was done using STATA software. To generate a clearer understanding of the relationship between IPEV and the explanatory variables, the data were analyzed at three levels which comprise univariate analysis where the study used weighted frequencies and percentages to show the distribution of each of the explanatory variables, namely, society-level factors, community-level factors, relationship-level factors, and individual-level factors.

The chi-square test of 95% confidence interval was used to determine the association between IPEV and women's empowerment (economic empowerment, attitudes justifying physical violence, and decision-making autonomy), partners' behaviors, and women's social demographic factors. The study used contingency tables in order to examine the relationship between IPEV and the explanatory variables.

The study conducted multivariable logistic regression analyses to assess predictors of IPEV. Results were reported using Odds Ratios (OR) at 95% confidence intervals. The binary logistic regression model was used where IPEV was modeled with background characteristics, attitudes justifying wife beating, and partners' controlling behavior. A link test was performed to determine the goodness of fit of the model.

### Ethical considerations

- The study ensured the confidentiality of the information extracted from the dataset.
- The authors also ensured that the dataset is strictly used for academic purposes and not any other role outside academics.
- The authors also sought permission from the supervisors to use the dataset. This involved the author clearly stating the study objectives in the proposal and, hence, the need to use the dataset.

## Results

### Background characteristics of the respondents

Table 1 shows the social demographic characteristics of the respondents which were assessed at the society, community, relationship, and individual levels. The majority (70%) of the respondents above the age of 25 years had experienced emotional violence and the least were in the age bracket of 15–24 years (30%). The majority were rural residents (78%). Most (82%) of the households were headed by male participants. Half (50%) of the women were married and half (50%) were cohabiting. More than half (59%) of the women had attained primary education and so were their partners (53%). Very few men and women had attained higher education (12 and 8%, respectively).

**Table 1:** Percentage distribution of married women who experienced IPEV by background characteristics.

**Social-demographic characteristics**	**Percentage (%)**	**Frequency**
**Age**		
15–24	29.7	1,675
25–34	38.0	2,145
35+	32.3	1,822
**Type of place of residence**		
Urban	22.3	1,261
Rural	77.7	4,382
**Sex of household head**		
Male	82.3	4,643
Female	17.7	1,000
**Current marital status**		
Married	50.0	2,820
Cohabiting	50.0	2,822
**Highest education level of women**		
No education	12.6	709
Primary	59.4	3,349
Secondary	20.4	1,153
Higher	7.6	431
**Partner's education level**		
No education	8.9	504
Primary	53.0	2,990
Secondary	26.1	1,472
Higher	12.0	677
**Number of other wives**		
No other wives	74.2	4,187
One or more co-wives	25.8	1,456
**Respondent's father ever beat her mother**		
No	64.1	3,614
Yes	35.9	2,028
**Type of earning's from respondent's work**		
Not paid	20.8	983
Cash only	46.9	2,212
Cash and in-kind	32.3	1,524
**Wealth index**		
Poorest	19.3	1,089
Poorer	20.6	1,160
Middle	20.1	1,135
Richer	18.8	1,059
Richest	21.3	1,200
**Ownership of assets**		
**(a) Owns land alone or jointly**		
No	57.2	3, 225
Yes	42.8	2,417
**(b) Owns a house alone or jointly**		
No	47.0	2,650
Yes	53.0	2,992
**Partner alcohol consumption**		
No	59.0	3,329
Yes	41.0	2,312
**Frequency of partner being drunk**		
Never gets drunk	11.5	265
Often gets drunk	32.2	744
Sometimes gets drunk	56.4	1,304
**Beating justified**		
No	50.1	2,819
Yes	49.9	2,823
Total	100	
**Decision making in household**		
No	12.8	724
Yes	87.2	4,919
**Partners' controlling behavior**		
No	30.8	1,735
Yes	69.2	3,907
Total	100	5,642

Some frequencies do not add up to 5,642 due to missing responses and/or filters that dropped some questions when a certain criterion was not met.

Almost three-quarters (74%) of the women had reported their husbands having no other wives whereas just over a quarter (26%) had reported their partners having at least one or more co-wives. Close to half (47%) earned cash only and 21% were not paid at all. Twenty-one percent of the women were from poorer and middle-wealth quintiles and more than one-third (36%) had witnessed parental violence. Half (50%) of the women had not been beaten by their partners but also half (50%) had justified being beaten for any of the reasons. The majority (87%) of the women participated in decision making and only 13% did not take active participation. Just over two-thirds (69%) of the men controlled their wives at home for any reason.

### Association of IPEV with women's background characteristics

Table 2 presents the association of IPEV with background characteristics. The results revealed that all the background factors were significantly associated with IPEV except the sex of the household head. IPEV was higher (82%) among women of 25 years and above who are rural residents (40%); 42% of the women are married and close to half (44%) are not educated. The least percentage has attained higher education (20%). A high percentage of their partners (42%) have not gone to school and 27% have attained higher education. Most women (44%) have reported their partners having no other wives and close to half (49%) have witnessed parental violence.

**Table 2:** Association of IPEV with women's background characteristics.

**Experienced IPEV in the last 12 months**				
**Variables**	**Yes (%)**	**No (%)**	**Frequency**	***p*** **-value**
**Age in 10-year groups**				
15–24	32.1	67.9	1,675	**0.000**
25–34	37.9	62.2	2,146	
35+	43.7	56.3	1,822	
**Type of place of residence**				
Urban	31.9	68.1	1,261	**0.000**
Rural	39.8	60.2	4,382	
**Sex of household head**				
Male	38.5	61.5	4,643	0.134
Female	35.6	64.4	999	
**Current marital status**				
Married	42.0	58.0	2,820	**0.000**
Cohabiting	34.1	65.9	2,822	
**Highest education level of wife**				
No education	44.0	56.0	709	**0.000**
Primary	41.7	58.3	3,349	
Secondary	30.2	69.8	1,153	
Higher	20.2	78.8	431	
**Husband/partner's education level**				
No education	42.3	57.7	504	**0.000**
Primary	42.3	57.7	2,990	
Secondary	33.2	66.8	1,472	
Higher	26.5	73.5	677	
**Number of other wives**				
No other wives	43.5	56.5	4,187	**0.000**
One or more co-wives	36.1	63.9	1,456	
**Respondent's father ever beat her mother**				
No	32.2	67.8	3,614	**0.000**
Yes	48.5	51.5	2,028	
**Type of earning's from respondent's work**				
Not paid	41.3	58.6	983	**0.040**
Cash only	37.4	62.6	2,212	
Cash and in-kind	42.3	57.7	1,524	
**Wealth index**				
Poorest	44.5	55.5	1,089	**0.000**
Poorer	40.3	59.7	1,160	
Middle	42.1	57.9	1,135	
Richer	37.2	62.8	1,059	
Richest	26.8	73.2	1,200	
**Ownership of assets**				
**(a) Owns land alone or jointly**				
No	35.7	64.3	3,225	**0.001**
Yes	41.1	58.9	2,417	
**(b) Owns a house alone or jointly**				
No	34.9	65.2	2,650	**0.000**
Yes	40.8	59.2	2,992	
**Alcohol consumption**				
**Husband /partner drinks alcohol**				
No	29.6	98.7	3,329	**0.000**
Yes	50.1	49.9	2,313	
**Frequency of partner being drunk**				
Never gets drunk	26.8	73.2	265	**0.000**
Often gets drunk	64.6	35.4	744	
Sometimes gets drunk	46.5	53.5	1,304	
**Beating justified**				
No	33.0	67.0	2,819	**0.000**
Yes	43.0	57.0	2,823	
**Decision making in a household**				
No	44.1	55.9	724	**0.0015**
Yes	37.1	62.9	4,919	
**Partners' controlling behavior**				
No	15.5	84.5	1,735	**0.000**
Yes	48.0	52.0	3,907	
Total	100	100	5,642	

The bold values indicate there exists a significant association between the dependent and the independent variables.

Financially, 41 and 42% are not paid or receive cash and in-kind, respectively. The majority of the women are from the poorest wealth quintile (45%) and only 27% are considered to be rich. Most (41%) of the women own houses and land jointly with their partners.

Half (50%) of the men drink alcohol and more often get drunk (65%). A small percentage (27%) never gets drunk. Women justified (43%) being beaten by their husbands for any of the reasons such as burning food, neglecting children, refusing to have sex, arguing with their husbands, and going anywhere without telling their husbands. Most of the women (44%) do not make joint decisions with their partners in the household and 48% have their partners control their behavior for any reason. Only 16% of women are not controlled by men in any way.

### Predictors of IPEV

Women aged 25 years and above had increased odds of experiencing IPEV compared to those aged 15–24 years (OR = 1.31; CI = 0.99–1.72) and women residing in rural areas had reduced odds of experiencing IPEV compared to those in the urban areas (OR = 0.68; CI = 0.48–0.99). Women who are cohabiting were less likely to experience IPEV compared to those who are married (OR = 0.74; CI = 0.59–0.92). Women who had witnessed parental violence were more likely to experience IPEV compared to those who did not (OR = 1.37; CI = 1.09–1.70). The predictors of IPEV are shown in Table 3 below.

**Table 3:** Predictors of IPEV.

**Variables**	**Odds ratio**	***p*-value**	**95% confidence interval**	
**Age in 10-year groups**				
15–24 (Rc)				
25–34	1.31	**0.050**	0.99	1.72
35+	1.44	**0.018**	1.06	1.95
**Type of place of residence**				
Urban (Rc)				
Rural	0.68	**0.041**	0.48	0.99
**Current marital status**				
Married (Rc)				
Living with partner/cohabiting	0.74	**0.007**	0.59	0.92
**Respondent's father ever beat her mother**				
No (Rc)				
Yes	1.37	**0.006**	1.09	1.70
**Highest education level of woman**				
No education (Rc)				
Primary	1.12	0.495	0.811	1.54
Secondary	1.05	0.835	0.671	1.64
Higher	0.51	**0.052**	0.26	1.00
**Partner's education level**				
No education (Rc)				
Primary	0.88	0.483	0.62	1.26
Secondary	0.87	0.520	0.58	1.32
Higher	1.25	0.436	0.71	2.18
**Number of other wives**				
One or more co-wives (Rc)				
No other wives	0.91	0.450	0.71	1.64
**Type of earning from respondent's work**				
Not paid (Rc)				
Cash only	1.20	0.233	0.89	1.62
Cash and in kind	1.32	0.065	0.98	1.77
**Wealth index**				
Poorest (Rc)				
Poorer	1.11	0.489	0.83	1.47
Middle	1.36	**0.050**	0.99	1.85
Richer	1.06	0.749	0.13	1.54
Richest	0.83	0.475	0.51	1.37
**Ownership of assets**				
**(a) Owns land alone or jointly**				
No (Rc)				
Yes	1.21	0.185	0.91	1.61
**(b) Owns a house alone or jointly**				
No (Rc)				
Yes	0.84	0.230	0.63	1.12
**Alcohol consumption**				
**Frequency of partner being drunk**				
Never gets drunk (Rc)				
often gets drunk	4.24	**0.000**	2.85	6.31
Sometimes gets drunk	2.41	**0.000**	1.66	3.49
**Decision making in a household**				
No (Rc)				
Yes	0.86	0.443	0.58	1.27
**Beating justified**				
No (Rc)				
Yes	1.16	0.162	0.94	1.44
**Partners' controlling behavior**				
No (Rc)				
Yes	4.32	**0.000**	3.33	5.60

The bold values indicate there exists a significant association between the dependent and the independent variables.

Women whose partners drank often or sometimes had increased odds of experiencing IPEV (OR = 4.24, CI = 2.85–6.31; OR = 2.41, CI = 1.66–3.49), respectively, compared to those who never got drunk. Conclusively, women whose partners controlled their behavior for any reason were more likely to experience IPEV compared to those whose husbands did not control their behavior.

Results also show that the wealth index except middle-income people, education level of wife and partner, type of earnings, justification for wife beating except occupation, and ownership of land and a house had no significant relationship with IPEV, but the rest were significant.

## Discussion

The objectives of the study were to investigate the association between attitude toward wife beating and intimate partner emotional violence, assess the relationship between partners' controlling behavior and intimate partner emotional violence, evaluate the relationship between social demographic factors and intimate partner emotional violence, and examine the relationship between women empowerment and IPEV.

The study revealed that age is one of the most influential demographic predictors of IPEV. Women aged 25 years and older had increased odds of experiencing IPEV compared to those aged 15–24 years. In Uganda, women at the age of 25 years are entering marriage and, therefore, begin to be exposed to emotional violence and the risk increases with knowledge of women's rights. It is also likely that as a woman progresses in age, she becomes more psychologically abused by the partner who may resort to marrying again whereas the previous woman has limited chances of getting another partner due to age, having children to care for, and respect for herself. The study results are in agreement with the studies conducted by Capaldi et al. (2012), Ismayilova and El-Bassel (2013), Karakurt and Silver (2013), Wandera et al. (2015), and Karamagi et al. (2006), which revealed that violence increases with age. The study contradicts the studies by Naved et al. (2017), Onanubi et al. (2017), and Puri et al. (2012), which showed that women below 25 years of age were more likely to be victims of violence as compared to women aged 25 years and above.

The study revealed that women residing in rural areas had reduced odds of experiencing IPEV compared to those in urban areas. This is because women who experience denial of basic human needs such as education, access to assets, power, and control of resources have limited ability to make decisions which exposes them to emotional abuse regardless of the residence where they come from. The findings are in agreement with the studies conducted by Osinde et al. (2011) and Ismayilova and El-Bassel (2013). However, study results contradict with results of other studies where emotional violence is high in rural areas (Bazargan-Hejazi et al., 2013; UBOS ICF, 2017). This is because a majority of these women in rural areas were illiterate, and majorly employed in subsistence agriculture which was characterized by low incomes, and hence cannot make any decisions at home and this makes them susceptible to emotional violence.

Education was another predictor of IPEV. Women with higher education levels were less likely to experience IPEV compared to those with no education. This is because high education levels among women make them more exposed, empowered economically, and have the higher bargaining power to decision-making compared to those who are not educated, and this decreases their risk to IPEV. The Uganda demographic survey from 2006 up to 2016 and the studies by Kwagala et al. (2013) reported similar findings.

Wealth status was another correlate of IPEV. Women who are middle-income earners were more likely to experience IPEV compared to those who are the poorest. This could be a result of failing to balance the responsibilities at home with work while earning so little to support the family. These results contradict the findings of studies conducted by Goodman et al. (2009), Vyas and Watts (2009), Osinde et al. (2011), Edwards et al. (2014), and Wandera et al. (2015) in Uganda and elsewhere.

In addition to the above, study results also show that women who are cohabiting had fewer odds to experience IPEV compared to those who are married. Women who do not stay with the child's father are likely to experience less emotional violence. This is in agreement with the study carried out by Huang et al. (2010). The study results contradict with a majority of the findings which stipulate that emotional violence is high among cohabiting couples (Capaldi et al., 2012; MacQuarrie et al., 2015; Bui et al., 2018).

Women who have witnessed parental violence were more likely to experience IPEV compared to those who did not. The environment in which we grow defines our behavior in the future. Witnessing violence during childhood teaches men that violence is an effective tool to resolve frustrations, stress, or conflict. It also teaches boys and men that violence is acceptable and appropriate to use to assert power. The women accept to be perpetrated by men because they have seen their fathers do the same to their mothers. The study findings are in agreement with the studies conducted by Karamagi et al. (2006), Speizer (2010), Wandera et al. (2015), and Kwagala and Wandera (2016) who suggested that witnessing violence as a child makes one become a perpetrator or a victim of violence in future.

Women whose partners drink often or sometimes had increased odds of experiencing IPEV. The study findings were expected because when men drink, they feel superior, their cognitive processing ability increases, impulse control is lowered, and information processing is distorted which sparks violence. Alcohol consumption makes victims violent, speak vulgar language, and men become aggressive which provokes quarrels and fights. This is in agreement with the studies conducted by Osinde et al. (2011), Devries et al. (2013), Ismayilova and El-Bassel (2013), and Wandera et al. (2015) who revealed that alcohol consumption was closely related to emotional violence.

Finally, study findings show that partners' controlling behavior had a significant relationship with IPEV. Results show that men who are jealous for reasons such as meeting friends without permission, husbands afraid of their partner most of the time, insisting to know where their wife is, and husbands jealous if a wife talked to other men had increased odds of experiencing IPEV compared to those who do not have any of the above characters. This is true because, in Uganda, most male participants dominate family structures where the woman is regarded as one of the male properties which encourages men to impose rules over women, hence causing violence. In addition, men want to impose their masculinity to be secure at home and they do this by controlling their partners at all levels. This is supported by Vung et al. (2008), Antai (2011), Durevall and Lindskog (2015), and Wandera et al. (2015), who revealed that partners' controlling behavior can induce emotional violence at home.

## Conclusion

We conclude that age, type of residence, current marital status, respondent's father ever beaten her mother, education, wealth quintile, drinking alcohol, and controlling behaviors influence IPEV. Women aged above 25 years were more likely to experience IPEV as compared to those aged below 25 years. With residence, women in rural areas were less likely to experience IPEV as compared to their counterparts in urban areas. Women with a higher level of education are less likely to experience IPEV compared to those with no education; women whose partners drink alcohol are more likely to experience IPEV compared to those who do not drink alcohol; women who are cohabiting are less likely to experience IPEV compared to those who are married; and women who witnessed parental violence were more likely to experience IPEV compared to those who never witnessed intimate partner emotional violence.

## Recommendations

Intimate partner emotional violence has been present since time immemorial with its roots largely in the patriarchal control of women by men and associated with lower status of women. The factors like partners' controlling behavior can be eliminated if IPEV eradication programs are designed to empower women, work with men to change their understanding of masculinity, and modify gendered institutions, policies, and laws toward achieving greater equality.

Education being a significant predictor of IPEV, there is a need to encourage girls to attain higher education so that they become empowered with knowledge and the ability to exercise their rights in a household. The government should also strengthen enforcement of alcohol-related laws and regulations and step up funding to the budgets of the sectors mandated to handle violence against women so that we do not depend only on donor aid because if they withdraw the funds then VAW programs will collapse.

Age being a significant predictor of IPEV, there is a need to promote healthy relationships among the younger age groups. This could be done through interventions such as encouraging teenagers to always have open discussions with friends of the opposite sex; forming clubs where teenagers and young people can openly talk about their relationships or sex life; and building self-esteem among the teenagers/adolescents so that they can make informed decisions regarding their lives.

In regard to the type of residence, there is a need to provide support and equipment to the health facilities in rural and urban areas. This is so because victims of IPEV seek treatment from health facilities. When health facilities are provided with the required equipment, they can easily identify victims, provide treatment, and refer victims who require specialized services.

Additionally, there is a need to focus on training healthcare providers to identify and respond to IPEV victims and drawing up guidelines for the proper management of IPEV victims.

Marital status being a significant predictor, there is a need for the married coordinating councils to monitor and exchange information about IPEV causes and effects on the married people in churches and communities. This will help to identify and address the problem.

Finally, there is also a need to carry out IPEV outreaches to the communities and prevention campaigns to raise awareness about the problem and to change social norms and behaviors that indirectly/directly lead to IPEV.

## Study limitations

Intimate partner emotional violence is self-reporting where any biases and errors may be due to privacy concerns and memory lapses which could have translated into an underestimation or overestimation of the prevalence of emotional violence.

The study used UDHS 2016 data, the researcher had no influence on the study design, sample size determination, data collection, and data entry which might have affected the results of the study.

## Data availability statement

Publicly available datasets were analyzed in this study. This data can be found here: www.ubos.org.

## Author contributions

RN conceived, designed, implemented the study inclusive of data analysis and presentation, interpretation, and discussion of results. AN provided guidance on study conceptualization, data analysis, and interpretation of results. SW guided the study conceptualization and advised on data analysis. All authors participated in drafting the manuscript and read and approved the final version.
